# *catena*-Poly[cobalt(II)-bis­(μ-3,7-dichloro­quinoline-8-carboxyl­ato-κ^3^ *N*,*O*:*O*′)]

**DOI:** 10.1107/S1600536807066755

**Published:** 2007-12-21

**Authors:** Zequan Li, Fengjing Wu, Yun Gong, Yunhuai Zhang, Chenguang Bai

**Affiliations:** a Department of Chemistry, College of Chemistry and Chemical Engineering, Chongqing University, Chongqing 400044, People’s Republic of China; b School of Materials Science and Engineering, Chongqing University, Chongqing 400044, People’s Republic of China

## Abstract

In the crystal structure of the title compound, [Co(C_10_H_4_Cl_2_NO_2_)_2_]_*n*_, the Co^II^ cation lies on a twofold rotation axis. Each cation is *N*,*O*-chelated by the carboxyl­ate anions of two 3,7-dichloro­quinoline-8-carboxyl­ate ligands. The second carboxyl­ate O atom of each ligand coordinates to the Co^II^ cation of an adjacent mol­ecule, linking the cations into a linear chain. Strong inter­chain π–π stacking inter­actions are observed in the crystal structure (perpendicular distance 3.42 Å, centroid-to-centroid distance 3.874 Å)

## Related literature

For the use of 3,7-dichloro-8-quinoline­carboxylic acid as a herbicide, see: Nuria *et al.* (1997 ▶); Pornprom *et al.* (2006 ▶); Sunohara & Matsumoto (2004 ▶); Tresch & Grossmann (2002 ▶). For related vanadium and cadmium complexes, see Chen *et al.* (2001 ▶); Yang *et al.* (2005 ▶). For related literature, see: Turel *et al.* (2004 ▶); Zhang *et al.* (2007 ▶).
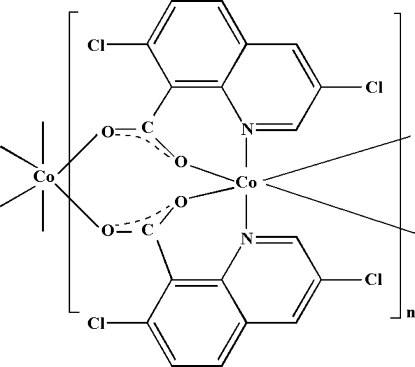


## Experimental

### 

#### Crystal data

[Co(C_10_H_4_Cl_2_NO_2_)_2_]
*M* *_r_* = 541.01
Orthorhombic, 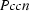

*a* = 13.5109 (14) Å
*b* = 15.964 (2) Å
*c* = 9.2157 (16) Å
*V* = 1987.7 (5) Å^3^
*Z* = 4
Mo *K*α radiation
μ = 1.43 mm^−1^
*T* = 298 (2) K
0.49 × 0.33 × 0.31 mm

#### Data collection

Siemens SMART CCD area-detector diffractometer
Absorption correction: multi-scan (*SADABS*; Sheldrick, 1996 ▶) *T*_min_ = 0.57, *T*_max_ = 0.64
9558 measured reflections
1752 independent reflections
1404 reflections with *I* > 2σ(*I*)
*R*_int_ = 0.039

#### Refinement

*R*[*F* ^2^ > 2σ(*F* ^2^)] = 0.034
*wR*(*F* ^2^) = 0.089
*S* = 1.11
1752 reflections
141 parameters
H-atom parameters constrained
Δρ_max_ = 0.67 e Å^−3^
Δρ_min_ = −0.76 e Å^−3^

### 

Data collection: *SMART* (Siemens, 1996 ▶); cell refinement: *SAINT* (Siemens, 1996 ▶); data reduction: *SAINT*; program(s) used to solve structure: *SHELXS97* (Sheldrick, 1997*a* ▶); program(s) used to refine structure: *SHELXL97* (Sheldrick, 1997*a* ▶); molecular graphics: *SHELXTL* (Sheldrick, 1997*b* ▶); software used to prepare material for publication: *SHELXTL*.

## Supplementary Material

Crystal structure: contains datablocks global, I. DOI: 10.1107/S1600536807066755/sj2456sup1.cif

Structure factors: contains datablocks I. DOI: 10.1107/S1600536807066755/sj2456Isup2.hkl

Additional supplementary materials:  crystallographic information; 3D view; checkCIF report

## Figures and Tables

**Table:** 

Co1—O1	2.093 (2)
Co1—O2^i^	2.057 (2)
Co1—N1	2.197 (2)

**Table:** 

O2^i^—Co1—O2^ii^	103.60 (12)
O2^i^—Co1—O1	170.96 (9)
O2^ii^—Co1—O1	85.43 (8)
O1—Co1—O1^iii^	85.55 (12)
O2^i^—Co1—N1	90.97 (9)
O2^ii^—Co1—N1	87.24 (9)
O1—Co1—N1	89.82 (9)
O1^iii^—Co1—N1	92.31 (9)
N1^iii^—Co1—N1	177.10 (14)

Symmetry codes: (i) 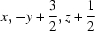; (ii) 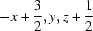; (iii) 
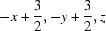
.

**Fig. 1.**
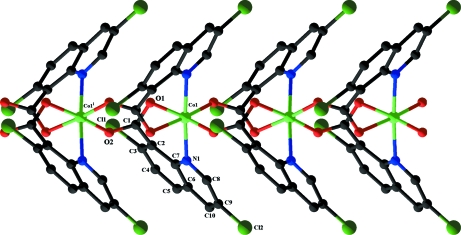
The structure of (I), with the atomic numbering scheme and displacement ellipsoids at the 50% probability level. H atoms have been omitted for clarity [Symmetry code: (i) x,-y + 1/2,z + 1/2.]

**Fig. 2.**
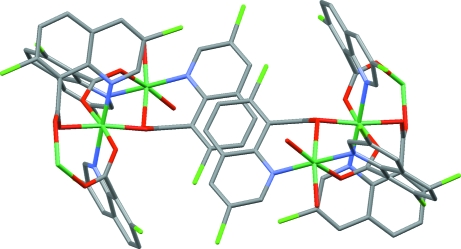
Three dimensional supramolecular architecture constructed by interchain π–π stacking interactions.

## supplementary crystallographic information

### Comment

Quinolinecarboxylates generally chelate to metal atoms, and some metal quinolinecarboxylates have been reported such as, for example, bis(6-methyl-4-hydroxy-3-quinolinecarboxylate) mono(oxo)monohydroxyvanadium(V) and Cd(H_2_O)(4-quinolinecarboxylato)_2_ (Chen *et al.*, 2001; Yang *et al.*, 2005). Quinclorac (3,7-dichloro-8-quinolinecarboxylic acid) is a most effective herbicides (Nuria *et al.*, 1997; Pornprom *et al.*, 2006; Sunohara & Matsumoto, 2004; Tresch & Grossmann, 2002). We have reported a nickel-quinclorac complex in our previous work (Zhang *et al.*, 2007). The title compound is a cobalt(II) derivative (I) (Fig. 1) with the Co^II^ cation located on a twofold rotation axis. The Co^II^ center exhibits a distorted octahedral geometry defined by four carboxylato oxygen atoms from four quinclorac and two nitrogen atoms from two quinclorac units. Each quinclorac ligand chelates to the cobalt atom *via* a quinoline N atom and a carboxylate O atom. Adjacent molecules are linked by carboxylate bridges into a linear chain. The chains are assembled into a three-dimensional supramolecular architecture by strong offset face-to-face π–π stacking interactions (perpendicular distance: 3.42 Å, centroid-centroid distance: 3.874 Å) between the C2–C7 and C2^i^–C7^i^ benzene rings [symmetry code: (i) 2 - *x*, 1 - *y*, - *z*].

### Experimental

A mixture of quinclorac (0.5 mmol, 0.121 g), CoCl_2_.6H_2_O (1 mmol, 0.238 g), Na_2_MoO_4_.2H_2_O (0.5 mmol, 0.121 g) and H_2_O (10 ml) was treated with aqueous HCl to a pH of 5. The mixture was placed in a Teflon-lined autoclave; this was heated at 403 K for three days. Red crystals were collected and washed with water. C H & N elemental analysis. Calculated for C_20_H_8_Cl_4_N_2_O_4_Co: C 44.36, H 1.48, N 5.18%; found: C 44.48, H 1.69, N 5.31%. Selected FT—IR (KBr, cm^-1^): 3301(w), 1581(*s*), 1553(*m*), 1482(*m*), 1402(*m*), 1383(*s*), 1232(*m*), 1139 (*m*), 1101(*s*), 761(*m*), 553(*m*), 449(*m*).

### Refinement

H atoms were positioned geometrically and refined as riding atoms, with C—H = 0.93Å and *U*_iso_(H) = 1.2*U*_eq_(C).

### Crystal data

**Table:**

[Co(C_10_H_4_Cl_2_NO_2_)_2_]	*F*_000_ = 1076
*M**_r_* = 541.01	*D*_x_ = 1.808 Mg m^−^^3^*D*_m_ = 1.800 Mg m^−^^3^*D*_m_ measured by not measured
Orthorhombic, *P**c**c**n*	Mo *K*α radiation λ = 0.71073 Å
Hall symbol: -P 2ab 2ac	Cell parameters from 9558 reflections
*a* = 13.5109 (14) Å	θ = 2.0–25.0º
*b* = 15.964 (2) Å	µ = 1.43 mm^−^^1^
*c* = 9.2157 (16) Å	*T* = 298 (2) K
*V* = 1987.7 (5) Å^3^	Block, red
*Z* = 4	0.49 × 0.33 × 0.31 mm

### Data collection

**Table:**

Siemens SMART CCD area-detector diffractometer	1752 independent reflections
Radiation source: fine-focus sealed tube	1404 reflections with *I* > 2σ(*I*)
Monochromator: graphite	*R*_int_ = 0.039
*T* = 298(2) K	θ_max_ = 25.0º
φ and ω scans	θ_min_ = 2.0º
Absorption correction: multi-scan(SADABS; Sheldrick, 1996)	*h* = −16→13
*T*_min_ = 0.57, *T*_max_ = 0.64	*k* = −18→18
9558 measured reflections	*l* = −10→9

### Refinement

**Table:**

Refinement on *F*^2^	Secondary atom site location: difference Fourier map
Least-squares matrix: full	Hydrogen site location: inferred from neighbouring sites
*R*[*F*^2^ > 2σ(*F*^2^)] = 0.034	H-atom parameters constrained
*wR*(*F*^2^) = 0.089	*w* = 1/[σ^2^(*F*_o_^2^) + (0.0291*P*)^2^ + 3.6236*P*] where *P* = (*F*_o_^2^ + 2*F*_c_^2^)/3
*S* = 1.11	(Δ/σ)_max_ = 0.001
1752 reflections	Δρ_max_ = 0.67 e Å^−^^3^
141 parameters	Δρ_min_ = −0.76 e Å^−^^3^
Primary atom site location: structure-invariant direct methods	Extinction correction: none

### Special details

**Table:**

Geometry. All e.s.d.'s (except the e.s.d. in the dihedral angle between two l.s. planes) are estimated using the full covariance matrix. The cell e.s.d.'s are taken into account individually in the estimation of e.s.d.'s in distances, angles and torsion angles; correlations between e.s.d.'s in cell parameters are only used when they are defined by crystal symmetry. An approximate (isotropic) treatment of cell e.s.d.'s is used for estimating e.s.d.'s involving l.s. planes.
Refinement. Refinement of *F*^2^ against ALL reflections. The weighted *R*-factor *wR* and goodness of fit *S* are based on *F*^2^, conventional *R*-factors *R* are based on *F*, with *F* set to zero for negative *F*^2^. The threshold expression of *F*^2^ > σ(*F*^2^) is used only for calculating *R*-factors(gt) *etc*. and is not relevant to the choice of reflections for refinement. *R*-factors based on *F*^2^ are statistically about twice as large as those based on *F*, and *R*- factors based on ALL data will be even larger.

### Fractional atomic coordinates and isotropic or equivalent isotropic displacement parameters (Å^2^)

**Table:**

	*x*	*y*	*z*	*U*_iso_*/*U*_eq_	
Co1	0.7500	0.7500	0.25905 (6)	0.02337 (17)	
Cl1	0.77826 (7)	0.45967 (6)	−0.08682 (10)	0.0438 (3)	
Cl2	1.11100 (8)	0.71113 (7)	0.54570 (14)	0.0641 (4)	
N1	0.89374 (19)	0.68581 (16)	0.2651 (3)	0.0271 (6)	
O1	0.70392 (16)	0.66997 (13)	0.0924 (2)	0.0296 (5)	
O2	0.80139 (16)	0.65857 (13)	−0.1029 (2)	0.0287 (5)	
C1	0.7764 (2)	0.64027 (19)	0.0240 (3)	0.0253 (7)	
C2	0.8430 (2)	0.57762 (19)	0.0989 (3)	0.0254 (7)	
C3	0.8519 (2)	0.4963 (2)	0.0541 (4)	0.0305 (7)	
C4	0.9198 (3)	0.4403 (2)	0.1170 (4)	0.0406 (9)	
H4	0.9214	0.3846	0.0871	0.049*	
C5	0.9831 (3)	0.4674 (2)	0.2212 (4)	0.0405 (9)	
H5	1.0300	0.4309	0.2594	0.049*	
C6	0.9783 (2)	0.5510 (2)	0.2723 (4)	0.0325 (8)	
C7	0.9051 (2)	0.60506 (19)	0.2140 (3)	0.0273 (7)	
C8	0.9570 (2)	0.7133 (2)	0.3621 (4)	0.0325 (8)	
H8	0.9503	0.7681	0.3949	0.039*	
C9	1.0338 (2)	0.6646 (2)	0.4188 (4)	0.0378 (8)	
C10	1.0438 (3)	0.5835 (2)	0.3773 (4)	0.0397 (9)	
H10	1.0929	0.5499	0.4175	0.048*	

### Atomic displacement parameters (Å^2^)

**Table:**

	*U*^11^	*U*^22^	*U*^33^	*U*^12^	*U*^13^	*U*^23^
Co1	0.0269 (3)	0.0247 (3)	0.0185 (3)	0.0027 (3)	0.000	0.000
Cl1	0.0472 (5)	0.0369 (5)	0.0472 (6)	−0.0042 (4)	−0.0028 (4)	−0.0106 (4)
Cl2	0.0561 (6)	0.0563 (7)	0.0799 (8)	0.0064 (5)	−0.0383 (6)	−0.0098 (6)
N1	0.0271 (14)	0.0274 (14)	0.0268 (15)	0.0037 (11)	−0.0010 (11)	0.0010 (11)
O1	0.0316 (12)	0.0338 (12)	0.0233 (12)	0.0062 (10)	−0.0014 (10)	−0.0035 (10)
O2	0.0344 (12)	0.0307 (12)	0.0211 (12)	0.0016 (10)	0.0015 (9)	0.0020 (10)
C1	0.0322 (17)	0.0217 (15)	0.0220 (16)	−0.0028 (12)	−0.0030 (13)	0.0000 (12)
C2	0.0258 (16)	0.0285 (16)	0.0218 (16)	0.0024 (13)	0.0053 (13)	0.0052 (13)
C3	0.0331 (17)	0.0261 (17)	0.0323 (18)	−0.0021 (14)	0.0046 (14)	0.0002 (14)
C4	0.051 (2)	0.0242 (18)	0.047 (2)	0.0059 (16)	0.0032 (19)	−0.0001 (16)
C5	0.042 (2)	0.0340 (19)	0.045 (2)	0.0122 (16)	−0.0004 (17)	0.0073 (17)
C6	0.0332 (18)	0.0330 (18)	0.0311 (19)	0.0051 (14)	0.0012 (14)	0.0049 (15)
C7	0.0266 (16)	0.0289 (17)	0.0264 (17)	0.0044 (13)	0.0070 (13)	0.0044 (14)
C8	0.0292 (17)	0.0315 (18)	0.037 (2)	0.0018 (14)	−0.0010 (15)	0.0008 (15)
C9	0.0306 (18)	0.042 (2)	0.041 (2)	0.0018 (15)	−0.0088 (16)	−0.0023 (17)
C10	0.0337 (19)	0.045 (2)	0.040 (2)	0.0106 (16)	−0.0065 (16)	0.0053 (18)

### Geometric parameters (Å, °)

**Table:**

Co1—O1	2.093 (2)	C2—C3	1.368 (4)
Co1—O1^i^	2.093 (2)	C2—C7	1.422 (4)
Co1—O2^ii^	2.057 (2)	C3—C4	1.406 (5)
Co1—O2^iii^	2.057 (2)	C4—C5	1.357 (5)
Co1—N1^i^	2.197 (2)	C4—H4	0.9300
Co1—N1	2.197 (2)	C5—C6	1.416 (5)
Cl1—C3	1.738 (3)	C5—H5	0.9300
Cl2—C9	1.734 (4)	C6—C10	1.410 (5)
N1—C8	1.312 (4)	C6—C7	1.419 (4)
N1—C7	1.381 (4)	C8—C9	1.398 (5)
O1—C1	1.257 (4)	C8—H8	0.9300
O2—C1	1.252 (4)	C9—C10	1.358 (5)
O2—Co1^iv^	2.057 (2)	C10—H10	0.9300
C1—C2	1.512 (4)		
			
O2^ii^—Co1—O2^iii^	103.60 (12)	C7—C2—C1	119.2 (3)
O2^ii^—Co1—O1	170.96 (9)	C2—C3—C4	122.5 (3)
O2^iii^—Co1—O1	85.43 (8)	C2—C3—Cl1	119.6 (3)
O2^ii^—Co1—O1^i^	85.43 (8)	C4—C3—Cl1	117.9 (3)
O2^iii^—Co1—O1^i^	170.96 (8)	C5—C4—C3	120.0 (3)
O1—Co1—O1^i^	85.55 (12)	C5—C4—H4	120.0
O2^ii^—Co1—N1^i^	87.24 (9)	C3—C4—H4	120.0
O2^iii^—Co1—N1^i^	90.97 (9)	C4—C5—C6	120.5 (3)
O1—Co1—N1^i^	92.31 (9)	C4—C5—H5	119.8
O1^i^—Co1—N1^i^	89.82 (9)	C6—C5—H5	119.8
O2^ii^—Co1—N1	90.97 (9)	C10—C6—C5	123.1 (3)
O2^iii^—Co1—N1	87.24 (9)	C10—C6—C7	118.3 (3)
O1—Co1—N1	89.82 (9)	C5—C6—C7	118.6 (3)
O1^i^—Co1—N1	92.31 (9)	N1—C7—C6	121.1 (3)
N1^i^—Co1—N1	177.10 (14)	N1—C7—C2	118.5 (3)
C8—N1—C7	118.2 (3)	C6—C7—C2	120.5 (3)
C8—N1—Co1	115.9 (2)	N1—C8—C9	123.5 (3)
C7—N1—Co1	121.7 (2)	N1—C8—H8	118.2
C1—O1—Co1	111.49 (19)	C9—C8—H8	118.2
C1—O2—Co1^iv^	130.7 (2)	C10—C9—C8	119.9 (3)
O2—C1—O1	126.2 (3)	C10—C9—Cl2	122.6 (3)
O2—C1—C2	114.9 (3)	C8—C9—Cl2	117.4 (3)
O1—C1—C2	119.0 (3)	C9—C10—C6	118.8 (3)
C3—C2—C7	117.8 (3)	C9—C10—H10	120.6
C3—C2—C1	122.9 (3)	C6—C10—H10	120.6
			
O2^ii^—Co1—N1—C8	9.9 (2)	Cl1—C3—C4—C5	176.0 (3)
O2^iii^—Co1—N1—C8	−93.6 (2)	C3—C4—C5—C6	3.0 (5)
O1—Co1—N1—C8	−179.1 (2)	C4—C5—C6—C10	−178.1 (4)
O1^i^—Co1—N1—C8	95.4 (2)	C4—C5—C6—C7	1.0 (5)
O2^ii^—Co1—N1—C7	166.4 (2)	C8—N1—C7—C6	4.7 (4)
O2^iii^—Co1—N1—C7	62.8 (2)	Co1—N1—C7—C6	−151.2 (2)
O1—Co1—N1—C7	−22.7 (2)	C8—N1—C7—C2	−174.0 (3)
O1^i^—Co1—N1—C7	−108.2 (2)	Co1—N1—C7—C2	30.1 (4)
O1^i^—Co1—O1—C1	68.44 (19)	C10—C6—C7—N1	−4.1 (5)
N1^i^—Co1—O1—C1	158.1 (2)	C5—C6—C7—N1	176.6 (3)
N1—Co1—O1—C1	−23.9 (2)	C10—C6—C7—C2	174.5 (3)
Co1^iv^—O2—C1—O1	8.1 (5)	C5—C6—C7—C2	−4.8 (5)
Co1^iv^—O2—C1—C2	−170.54 (19)	C3—C2—C7—N1	−177.0 (3)
Co1—O1—C1—O2	−109.2 (3)	C1—C2—C7—N1	7.7 (4)
Co1—O1—C1—C2	69.4 (3)	C3—C2—C7—C6	4.4 (4)
O2—C1—C2—C3	−65.8 (4)	C1—C2—C7—C6	−170.9 (3)
O1—C1—C2—C3	115.4 (3)	C7—N1—C8—C9	−1.4 (5)
O2—C1—C2—C7	109.2 (3)	Co1—N1—C8—C9	155.8 (3)
O1—C1—C2—C7	−69.6 (4)	N1—C8—C9—C10	−2.3 (6)
C7—C2—C3—C4	−0.3 (5)	N1—C8—C9—Cl2	179.7 (3)
C1—C2—C3—C4	174.8 (3)	C8—C9—C10—C6	2.8 (6)
C7—C2—C3—Cl1	−179.7 (2)	Cl2—C9—C10—C6	−179.4 (3)
C1—C2—C3—Cl1	−4.6 (4)	C5—C6—C10—C9	179.5 (4)
C2—C3—C4—C5	−3.4 (5)	C7—C6—C10—C9	0.4 (5)

Symmetry codes: (i) −*x*+3/2, −*y*+3/2, *z*; (ii) *x*, −*y*+3/2, *z*+1/2; (iii) −*x*+3/2, *y*, *z*+1/2; (iv) *x*, −*y*+3/2, *z*−1/2.
